# The political economy of health in the Gaza strip: Reversing de-development

**DOI:** 10.7189/jogh.12.03014

**Published:** 2022-04-02

**Authors:** Mona Jebril, Simon Deakin

**Affiliations:** Centre for Business Research, Judge Business School, University of Cambridge, Cambridge, UK

While the health sector in Gaza is affected by de-development, there are practical steps that can be taken to address the problem: hence our focus on ‘reversing de-development’. We draw on 14 in-depth semi-structured interviews conducted with policy makers and health officials, as well as people caring for patients in the Gaza Strip, which are reported at greater length in Jebril (2021) [1].

## THE DE-DEVELOPMENT OF THE GAZA STRIP

Sustainable development requires a certain level of state capacity. In situations where the state is financially insecure and administratively disorganised, its ability to deliver public goods, including health, is compromised. In Gaza’s case, the state is not just weak; its capacity is eroding over time. This feature, known as ‘de-development’ [2], is having seriously negative repercussions for Gaza’s health sector.

Gaza’s de-development is the result of several related factors. Decades of Israeli occupation has led to territorial, economic and political fragmentation [3]. The closure of Gaza’s borders resulted in a deformalized economy characterized by high levels of unemployment and precarious work, eroding the local tax base and increasing the government’s dependence on donor aid [4]. The Hamas takeover led to ‘sweeping, punitive sanctions against an entity and a population already exhibiting signs of severe political, social and economic stress’ [4]. It also widened the gap between the different parts of the Occupied Palestinian Territory (OPT), creating ‘two de facto governments in Ramallah and Gaza with parallel Ministries’ [5]. In the context of growing factionalism between Hamas and Fatah, public administration has become increasingly politicised. With funding insecure, wages of government workers are often left unpaid for months on end. ‘Institutional decline and degraded governance’ have created the conditions for ‘state collapse’ to an extent which is close to being ‘irreversible’ [4].

## HEALTH IN A CONTEXT OF DE-DEVELOPMENT

De-development is manifested in several ways in the health sector. Firstly, structures are fragmented. There are four main providers: UNRWA, Health NGOs, the Palestinian health ministry/ies, and the private sector. In addition, specialized tertiary health care is only available by transfers to Israel or neighbouring Arab countries [5]. Roles are not clearly defined, and responsibilities overlap, leading to duplication on the one hand and gaps in provision on the other [1]. The multiplicity of donors, and the split between Hamas and the Fatah-dominated Palestinian National Authority (PNA), have contributed significantly to this problem. For example, a senior policy maker (Interviewee2, AC1) commented: “we have to blame both parties, Hamas and Fatah because they are responsible for the division of the Palestinian people, and the duplication of services”.

**Figure:**
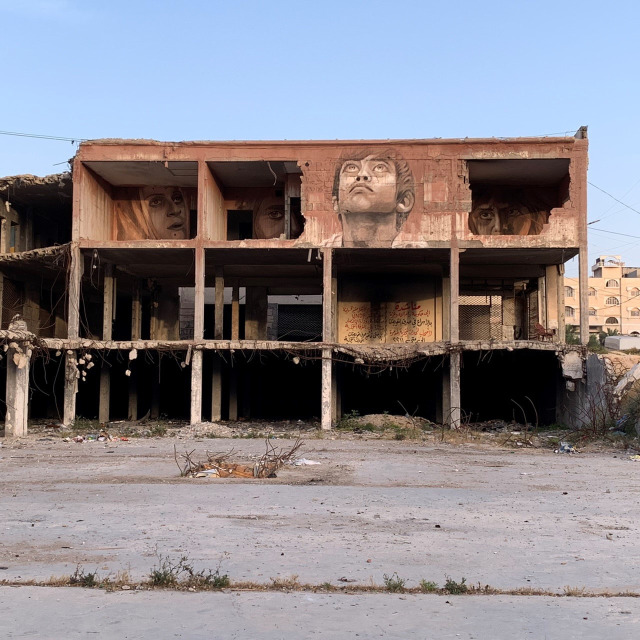
Photo: Mural by Ali Jabali, Gaza City, Photograph by Mona Kreigler, 2008/19).

Secondly, factionalism and political competition impede service delivery [1,6,7]. Factional nepotism in appointments and promotions is widely reported [1]. In addition, health is used as a political tool. The PNA, based in the West Bank, has used health to pressurize the Hamas government in Gaza, reducing financial approvals and restricting the supply of drugs between the West Bank and Gaza [1,5]. The sector is already affected by shortages of supply due to the prolonged Israeli and Egyptian blockade of Gaza’s borders [8]. According to the World Bank (2019), Israel also continues to put restrictions on an expanded list of “key production inputs, namely those deemed as ‘dual use’” [9]. This affects the availability of drugs for NCD patients, as well as limiting the maintenance capacity of equipment. The WHO (2017) reports that spare parts for electricity generators are among the items which have been restricted [10]. Since Gaza is affected by daily power cuts, this negatively impacts the service delivery capacity of hospitals.

Thirdly, reliance on donor aid heightens resource competition and increases coordination costs [11,12]. International assistance from agencies and NGOs is double edged: while it provides crucial services, technical help and life-saving support, it risks stymying the emergence of local service delivery. The Hamas-run health ministry relies significantly on donor support for covering the majority of day to day operations [5]; this support is precarious and coordination with donors is complex, occasionally flaring into crisis [5]. The government cannot compel foreign donors to commit to a particular project or always to see it through [13]. Donor priorities may not always be a good fit with basic needs: there is a tendency for NGOs to favour ‘grand projects’ related to ‘empowerment and civil society rather than focusing on serving the needs of the local population’ [11]. A senior policy maker, from the NGO sector commented: “This has negative consequences. We are living under the mercy of the funder, who is at the end serving his own agenda” [Interviewee 5, NGO1].

Fourthly, in the absence of a fully functioning state, kinship and social solidarity serve as substitute modes of coordination [1]. While often effective at more than a rudimentary level, they can operate in an exclusionary way, reinforcing social hierarchies and divisions which result in under-provision for certain groups [1]. For example, the majority of the interviewees reported that ‘wasta’ practices, which operate through kinship and social acquaintances, helped people to jump queues and receive improved medical care. ‘Wasta’ may be indispensable for those who can access it but not everyone is well connected, so its use reinforces inequalities [14]. Factionalism is reflected in clientelism, which is widely reported to affect the quality of recruitment into the health sector [7,15]. Patriarchal practices are widespread and give rise to stereotypical assumptions of women’s capacity for leadership in the field, and to females’ self-exclusion from roles that are traditionally assigned to their male counterparts [14,16]. For example, a health official (interviewee 8, NGO2) explained:

“If I have any objection, my boss will be standing [facing] me as if he is my father, […] and he may even threaten me. Although I work on an international project, the institution administration is Palestinian and [factional…]. Sometimes there is oppression or maybe arrogance”.

Another health official (Interviewee 6, IO3) told us:

“As females, we suffer when we go for a meeting with a male colleague. People will refer to us as the ‘ones with him', even if we are all doing the same thing”.

Our interview data show, nonetheless, that at times of emergency, these inequalities and hierarchies seem to wane. Feelings of ‘chivalry’, ‘a situational sense’ and a ‘religious duty’ help to mobilise a community-level response at times of crisis [1]. For example, a health official (Interviewee 11, PI2) explained:

“Political divisions disappear [when there is an emergency]. During the 2008 war on Gaza, [..] I saw people [in the hospital] donating blood, and others giving first aid, or helping as doctors. All these were individual initiatives”.

## DECISION MAKING AND THE CHALLENGES OF IMPLEMENTATION

In this environment, decision making is affected in various ways. The Hamas government and the PNA both lack sovereignty over borders and are denied essential resources [15]. The Palestinian schism has complicated things further, increasing the health sector’s politicisation, and undermining health management cooperation between the Gaza Strip and Ramallah [1,5]. This makes it almost impossible to have a unified strategic plan for the health sector; often, cooperation initiatives remain *‘ink on paper’* (Interviewee 5, NGO1). Consequently, decision making and implementation in the health sector are forced to rely on ad hoc judgements and interventions [13]. This leads to a top-down and authoritarian approach to identifying priorities and a lack of transparency. Creating and sharing data among the providers of health in the Gaza Strip is a major challenge, in part because Gaza lacks a modern health information system [13]. All this results in “serious structural and systematic problems that come in the way of turning plans into successful realities” [17]. The non-functioning of the Palestinian Legislative Council since 2007, a result of the split between Hamas and the Fatah dominated PNA, has created a policy vacuum in the Gaza health sector, retarding government’s ability to update standards, enforce accountability, and monitor quality of service delivery [1]. It also weakens the sector’s legal base, which is particularly problematic in face of the politicisation of the health and competition between the agendas of different funders.

## LIMITS OF A ‘MIXING APPROACH’ TO REFORM

The urgency of the situation on the ground in the Gaza Strip requires short-term humanitarian responses to continue alongside the long-term objectives of working towards peace and development. Thus reform “polices need to be guided by the principle of mixing selected possible and realizable reform measures with relief and emergency operations, rehabilitation and reconstruction” [17]. At the same time, although a mixing approach may help to meet short-term needs, focusing on immediate crises “has contributed to developing neither the infrastructure, nor the economy nor self-sustaining institutions” [1,3].

## WAYS FORWARD

It may be thought that reversing the downward trend of de-development in the Gaza Strip is impossible without a political solution, but this has been decades in the waiting already. In the meantime, there are practical steps that can be taken. Firstly, moves can be made at international level to recognise the health sector in Gaza as a humanitarian domain, preserving the Gazan people’s right to health at all times, for example, ensuring cancer patients’ immediate access to necessary drugs and treatment under conditions of blockade and providing sufficient international protection for health personnel and facilities, under conditions of war and internal conflict. Secondly, improving information flows, by building collaborations between stakeholders, would enable the more effective mobilization of the community’s scarce resources, as well as allowing health providers to work more effectively together to meet shared goals. Thirdly, uniting the Palestinian governments’ ministries of health would make it possible to articulate a national vision for reform and make it more straightforward for donor assistance to be directed to meeting local needs. Fourthly, advances in cloud computing and data mining should be explored for their potential in supporting health capacity, while bearing in mind the need to take account of ethical considerations and security concerns. Fifthly, updating the legal framework for the Gazan health sector would increase accountability and transparency among health providers, promote trust in the governance of the sector, and advance professionalism in face of social and partisan agendas.

As we make these recommendations, we recognize that they are not easy to implement, given that the Gaza Strip remains a politicized context, and one of ‘continuous suffering and emergency’ [1]. Nonetheless, we think that it is imperative that all providers and supporters of health care in Gaza, from both the local and international community, push in the direction of these reforms to the best of their capacity and build incrementally on the progress already being made. The alternative is to let the downward trend of de-development in the Gaza Strip deteriorate until it becomes irreparable. This would endanger the health of future generations of Palestinians and make it more difficult to achieve a durable peace. It would render futile attempts to realise the UN’s sustainable development goals in the Gazan context. We suggest that the potential for achieving reform does exist even under the current challenging circumstances of Gaza. The sector can start with a focus on human capacity building, the rationalization of health services, and working on health-related advocacy to achieve the recommendations we have set out. From our interviews [1], we can see that some are already working in this direction.
